# Exercise Questions on Chapter Fourth

**Published:** 1883-10

**Authors:** 


### EXERCISE QUESTIONS ON CHAPTER FOURTH.

What characteristics of chlorine give it its name?

Give the names of the other members of the chlorine group?

How are they closely related?

What is their group name?

Give the history of chlorine?

What is its symbol?

Give its occurrence?

What is its source in the laboratory?

Give the method of its preparation?

Describe the method of collecting a gas by displacement?

Can this method be used for a gas lighter than air?

What is the hydrochloric acid method of obtaining chlorine?

Give the physical properties of chlorine?

Its chemical properties?

What is its atomic weight?

What is its specific gravity?

Its quantivalece?

Give the chief characteristics of chlorine?

Why is it a disinfectant and deodorant?

Is it a septic or antiseptic?

Give the true difference between a disinfectant and an antiseptic?

Why does chlorine bleach?

What is the only compound of hydrogen and chlorine?

How is HCL prepared?

Is the gas an acid?

What is common hydrochloric acid?

How is it prepared?

Define the name of nitrogen?

Give its history?

Give the method of experimentation which led to its discovery?

What is its occurrence?

How is it prepared?

What substance is produced by the union of phosphorus and oxygen?

Is it a gas or a solid?

What becomes of it in the jar?

Give the physical properties of Nitrogen?

Its chemical properties?

